# Effect of metabolic syndrome on coronary heart disease in rural minorities of Xinjiang: a retrospective cohort study

**DOI:** 10.1186/s12889-020-08612-w

**Published:** 2020-04-25

**Authors:** Changjing Li, Jia He, Bin Wei, Xianghui Zhang, Xinping Wang, Jingyu Zhang, Kui Wang, Yunhua Hu, Lati Mu, Yizhong Yan, Jiaolong Ma, Yanpeng Song, Heng Guo, Rulin Ma, Shuxia Guo

**Affiliations:** Department of Public Health, Shihezi University School of Medicine, Shihezi, China

**Keywords:** Coronary heart disease, Metabolic syndrome, Interaction, Risk factor, Epidemiology

## Abstract

**Background:**

Metabolic syndrome is diagnosed by a cluster of risk factors that associated with an increased risk of coronary heart disease (CHD). We aimed to explore the impact of and interactions between individual metabolic syndrome components on the risk of CHD in Xinjiang.

**Methods:**

The baseline population included 7635 participants. The degree to which the components increase the risk of CHD and the multiplicative interactions between them were assessed using hazard ratios (HRs) and 95% confidence intervals (CIs). Additive interactions were appraised by the relative excess risk due to interaction, synergy index (SI), and attributable proportion of interaction.

**Results:**

A total of 304 CHD patients were enrolled from rural residents of Xinjiang. Elevated blood pressure (HR 1.81; 95% CI 1.35–2.44) and elevated fasting blood glucose (FBG) (HR 1.82; 95% CI 1.38–2.38) increased the risk of CHD after adjustment for confounding factors. We found a positive additive interaction (SI 1.14; 95% CI 0.51–2.51) between elevated blood pressure and elevated FBG, but none were significant. As the number of components increased, the risk of CHD increased. The combinations of [high triglycerides (TG) + low high-density lipoprotein cholesterol (HDL-C) + elevated FBG + *large* waistline] (HR 4.26; 95% CI 1.43–12.73) and [elevated blood pressure + elevated FBG + low HDL-C + *large* waistline] (HR 1.82; 95% CI 1.38–2.38) increased the risk of CHD.

**Conclusions:**

We provide evidence that elevated blood pressure and elevated FBG are independent risk factors for CHD and it might be necessary to maintain the normal waistline for preventing CHD.

## Background

The mortality rate of CHD has increased by 40.1% from 2006 to 2016, with CHD accounting for 17.8% of total deaths in China [1]. Meanwhile, the prevalence of risk factors for CHD has been increasing. CHD cannot be completely cured by clinical treatment, but it can be effectively prevented by controlling its risk factors and treating diseases known to contribute to this propensity. *Metabolic syndrome* is diagnosed by a range of components, including obesity, elevated blood pressure, elevated serum triglycerides, low *HDL-C*, and elevated fasting blood glucose [2, 3].

*Metabolic syndrome* is associated with an increased risk of CHD [4, 5], and a meta-analysis report indicates that the hazard ratio of CHD events in patients with *metabolic syndrome* is 1.65 (1.37–1.99), while the HR of CHD deaths is 1.60 (1.28–2.01) [6]. In addition, studies have shown that the contribution of *metabolic syndrome* and its components to the risk of CHD should be treated equally, and that their combined effect may have a stronger impact on CHD [7–9]. Another study found that the risk of CHD increases with the number of components [10]. However, the above research needs to be further confirmed in the Xinjiang population.

Ethnic minorities account for 64.90% of Xinjiang’s total population, and the Han community accounts for 35.10% of Xinjiang’s total population. However, there are less research on ethnic minorities. Therefore, we choose to analyze the major ethnic minority population in Xinjiang (the top two ethnic minority groups in Xinjiang: Uygur 73.83%, Kazakh 13.51%) [11]. Due to lack of economic development, difficult living environment, and specific diet (high salt, high fat, high carbohydrates) [12], the prevalence of *metabolic syndrome* among the population of Xinjiang is higher than that among the national population in China [13]. Moreover, as metabolic syndrome component indicators are simple to obtain, it is essential to analyze the effect of metabolic syndrome on coronary heart disease in this population.

In the present study, we analyzed the aggregation of its components and the effects of interactions between *metabolic syndrome* components on the risk of CHD in this population, which can be instrumental in the prevention and control of CHD.

## Methods

### Study Population

The survey among Kazakhs and Uyghurs began in April 2009 and was followed up in December 2013, April 2016, and August 2017 in Xinyuan County and Jiashi County. We used a stratified sampling method to select the corresponding villages (9 villages in the Halabra Township, 6 villages in the Nalati Township, and 12 villages in the Jiangbazi Township). We followed 7635 participants (3546 Uighurs, 4089 Kazakhs) above the age of 18 years who had lived in the villages for at least 6 months. All participants provided informed consent. The total response rate was 87.5% (88.6% in Uygur and 86.5% in Kazakh). We further excluded 133 participants who already had CHD before April 2009 and 220 participants with incomplete blood samples or physical information. Finally, we included 7282 participants in this study. The survey was approved by the Ethical Review Board of the First Affiliated Hospital of Shihezi University School of Medicine (IERB No. SHZ2010LL01) and operations and methods were carried out in accordance with the relevant guidelines and regulations.

### Diagnostic criteria for CHD

Patients diagnosed with CHD needed to meet the criteria for having their first CHD hospitalizations during the study period due to one or more of the following: coronary artery atherosclerosis, coronary interventional therapy, angina pectoris, myocardial infarction, and sudden cardiac death. The patients of CHD were determined based on self-reported questionnaire responses, medical insurance records, and local hospital discharge records from 2009 to 2017. Patients with self-reported manifestations of CHD findings required a certificate of diagnosis from a medical institution in their township during the investigation.

### Epidemiological survey and biochemical measurements

Data of participants were collected using a questionnaire. The detailed questions on the questionnaire covered demographics, smoking status, alcohol consumption status, and personal and family history of disease. During the interview, each participant’ s *waistline* was measured by uniform standardized methods. The above measurement and blood sample collection methods have been previously described [14]. The biochemical parameters from blood samples included TG, high-density lipoprotein cholesterol, and FBG, which were analyzed using an automatic biochemical analyzer (Olympus AU 2700; Olympus Diagnostics, Hamburg, Germany).

### Definition of metabolic syndrome

Our study used the National Cholesterol Education Program (NCEP) definition for diagnosis of *metabolic syndrome* in order to achieve the goal of controlling high-risk populations to reduce development of CHD [15]. The individuals were diagnosed as having *metabolic syndrome* if 3 or more of the 5 components were present, as follows: (1) *waistline* (≥ 90 cm in men ≥80 cm in women); (2) FBG ≥ 5.6 mmol/L or a diagnosis of diabetes; (3) systolic blood pressure (SBP) ≥ 130 mmHg; diastolic blood pressure (DBP) ≥ 85 mmHg, or use of anti-hypertensive drugs; (4) high TG (≥ 1.7 mmol/L); and (5) low HDL-C cholesterol (< 1.04 mmol/L in men and < 1.30 mmol/L in women) [2].

### Statistical analysis

Significant differences in baseline characteristics were found using the χ^2^ test and t test. Evaluation of association between *metabolic syndrome* components and CHD risk was assessed using multivariate Cox regression. The above multivariable Cox model were tested by the proportional hazard assumption of Cox models. Data were analyzed using Statistical Product and Service Solutions (SPSS) version 24.0 (Chicago, Illinois, USA). Statistical significance was defined as a *p*-value< 0.05 and all statistical tests were two-sided.

Multiplicative interactions were evaluated by the multiplicative SI and its 95% CI (the 95%CI of multiplicative SI not including 1 indicate significant multiplicative interaction), which was calculated by entering the two risk factors and their interaction terms into the Cox regression. We appraised additive interactions by 95% CI of three indices; the following conditions indicate exist additive interaction: relative excess risk due to interaction (RERI) not including 0, attributable proportion of interaction (AP) not including 0, and additive SI not including 1, which were calculated using the Andersson’s calculation table. RERI is used to describe the magnitude of the risk attributed to the interaction, which is the absolute value of the difference between the combined effect of two factors and the sum of its individual effects. RERI = 0 means there is no additive interaction; the larger the absolute value of RERI, the stronger interaction between factors. AP means the proportion of the total risk of the disease attributable to its interaction. When SI = 1 means there is no interaction and two factors are independent of each other; SI > 1 means there is a positive interaction; SI < 1 means there is a negative interaction [16].

## Results

### Baseline characteristics of the study participants

In this study, 7282 people completed the follow-up and the average follow-up time was 6.501 ± 2.834 years (the follow-up time range was 1.003–8.708 years). We identified 304 patients (63.48% for female, average age 55.23 ± 12.02 years old) having their first CHD event during the follow-up period, including three patients were under 30 years old. The incidence density of CHD was 6.42/1000 person-years (304/47349.16 person-years). The prevalence of metabolic syndrome was 26.96% (1963/7282) in the baseline population. Table 1 shows that in addition to family history of CHD, the other characteristics showed significant differences between the two groups (*p* < 0.05).

**Table 1:** Baseline Characteristics of the Study Participants

Characteristics	Without metabolic syndrome (*n* = 5319)	With metabolic syndrome (*n* = 1963)	*p*
Age (year)	38.19 ± 14.50	46.32 ± 13.44	< 0.001
Sex (Male), n (%)	2587 (48.64)	773 (39.38)	< 0.001
Smoking status, n (%)	1228 (23.09)	568 (28.94)	< 0.001
Alcohol consumption, n (%)	348 (6.54)	155 (7.90)	0.043
Family history of CHD, n (%)	321 (6.03)	131 (6.67)	0.316
Family history of diabetes, n (%)	75 (1.41)	42 (2.14)	0.028
Family history of hypertension, n (%)	1121 (21.08)	487 (24.81)	0.001
Incidence of CHD, n (%)	156 (2.93)	148 (7.54)	< 0.001

*CHD* coronary heart disease

### Association between the metabolic syndrome components and CHD

Table 2 shows that the risk of CHD increased significantly and was associated with metabolic syndrome (adjusted HR 1.81; 95% CI 1.44–2.28), elevated blood pressure (adjusted HR 1.81; 95% CI 1.35–2.44), and high FBG (adjusted HR 1.82; 95% CI 1.38–2.38), however, there were no significant differences were found for high TG and low HDL-C (*p* < 0.05).

**Table 2:** The association between metabolic syndrome components and coronary heart disease

Components	N	CHD, n (%)	HR ^a^ (95% CI)	HR ^b^ (95% CI)	HR ^c^ (95% CI)
*Large waistline*	3583	202 (66.45)	2.23 (1.76–2.84)	1.42 (1.10–1.83)	1.28 (0.99–1.66)
High TG	1382	73 (24.01)	1.45 (1.11–1.89)	1.26 (0.97–1.64)	1.08 (0.82–1.41)
Low HDL-C	3661	152 (50.00)	1.04 (0.83–1.31)	1.07 (0.85–1.34)	1.07 (0.85–1.35)
Elevated blood pressure	3821	242 (79.61)	3.73 (2.82–4.93)	1.92 (1.44–2.58)	1.81 (1.35–2.44)
Elevated FBG	850	72 (23.68)	2.78 (2.14–3.63)	1.94 (1.49–2.54)	1.82 (1.38–2.38)
*Metabolic syndrome*	1963	148 (48.68)	2.99 (2.39–3.75)	1.81 (1.44–2.28)	–

*FBG* fasting blood glucose*, HDL-C* high-density lipoprotein cholesterol*, TG* triglycerides*, CHD* coronary heart disease*, HR* hazard ratios*, CI* confidence interval*.* Large w*aistline* ≥ 90/80 cm; high TG ≥ 1.7 mmol/L; low HDL-C < 1.04/1.30 mmol/L; elevated blood pressure ≥ 130/85 mmHg; elevated FBG ≥ 5.6 mmol/L.

^a^ Without adjustment

^b^ Adjustment for alcohol consumption, smoking status, age, sex and family history of hypertension, family history of diabetes and family history of coronary heart disease

^c^ Adjustment for alcohol consumption, smoking status, age, sex and family history of hypertension, family history of diabetes and family history of coronary heart disease, and the other risk factors (i.e., large waistline, high TG, low HDL-C, elevated blood pressure and elevated FBG)

### Interactions among components of metabolic syndrome on the risk of CHD

The multiplicative interactions of elevated FBG and elevated blood pressure, and the effects of their coexistence effects with other components on the risk of CHD are shown in Table 3. The cumulative effect of elevated FBG and elevated blood pressure increased the risk of CHD (adjusted HR 3.51; 95%CI 2.37–5.19). The cumulative effects between other components and elevated FBG increased the risk of CHD (adjusted HR 3.97; 95%CI 2.42–6.51). The cumulative effects between other components and elevated blood pressure increased the risk of CHD (adjusted HR 3.58; 95%CI 2.49–5.15). However, the multiplicative interaction was not significant between elevated FBG and elevated blood pressure (multiplicative SI 0.79; 95%CI 0.39–1.58), elevated blood pressure and other components (multiplication SI 1.50; 95% CI 0.76–2.77), elevated FBG and other components (multiplication SI 1.91; 95% CI 0.67–3.43).

**Table 3:** Multiplicative interactions among components of *metabolic syndrome* on the risk of coronary heart disease

Interaction items	N	CHD, n (%)	HR ^b^ (95% CI)	Multiplicative SI ^b^ (95%CI)
Elevated FBG ^−^ / Elevated blood pressure ^−^	3168	50 (1.58)	1 (ref)	0.79 (0.39–1.58)
Elevated FBG ^+^ / Elevated blood pressure ^−^	3264	182 (5.58)	1.95 (1.41–2.70)	
Elevated FBG ^−^ / Elevated blood pressure ^+^	293	12 (4.1)	2.29 (1.22–4.30)	
Elevated FBG ^+^ / Elevated blood pressure ^+^	557	60 (10.77)	3.51 (2.37–5.19)	
Elevated blood pressure ^−^/ Other components ^−^	3100	49 (1.58)	1 (ref)	1.50 (0.76–2.77)
Elevated blood pressure ^+^/ Other components ^−^	2464	159 (6.45)	2.65 (1.51–4.63)	
Elevated blood pressure ^−^/ Other components ^+^	299	13 (4.35)	1.00 (0.57–1.75)	
Elevated blood pressure ^+^/ Other components ^+^	1115	83 (7.44)	3.97 (2.42–6.51)	
Elevated FBG ^−^/ Other components ^−^	4896	144 (2.94)	1 (ref)	1.91 (0.67–3.43)
Elevated FBG ^+^/ Other components ^−^	668	64 (9.58)	1.55 (0.74–3.25)	
Elevated FBG ^−^/ Other components ^+^	1304	88 (6.75)	1.21 (0.90–1.65)	
Elevated FBG ^+^/ Other components ^+^	110	8 (7.27)	3.58 (2.49–5.15)	

*FBG* fasting blood glucose, *CHD* coronary heart disease, *HR* hazard ratio, *CI* confidence interval. Other components, one or more components are abnormal among the three components except blood sugar and blood pressure (large waistline, high TG, low HDL-C)

N is the number of participants in the interaction items. ^b^ Adjustment for alcohol consumption, smoking status, age, sex and family history of hypertension, family history of diabetes and family history of coronary heart disease

The following three groups of factors had positive additive interactions for CHD when they coexist, but none had statistical significance: elevated FBG and elevated blood pressure (RERI 0.25, 95%CI -1.23-1.73; AP 0.08, 95%CI -0.39-0.56; additive SI 1.14, 95%CI 0.51–2.51), elevated blood pressure and other components (RERI 0.344, 95%CI -0.28-0.97; AP 0.23, 95%CI -0.25-0.72; additive SI 3.54, 95%CI 0.01–3.68), elevated FBG and other components (RERI 0.63, 95%CI -0.34-1.60; AP 0.38, 95%CI -0.19-0.94; additive SI 22.94, 95%CI 0.001–17.99) (Table 4).

**Table 4:** Additive interactions among components of metabolic syndrome on the risk of coronary heart disease

Interactive items	Additive interaction		
	RERI ^b^ (95% CI)	AP ^b^ (95% CI)	SI ^b^ (95% CI)
Elevated FBG & Elevated blood pressure	0.25 (−1.23–1.73)	0.08 (−0.39–0.56)	1.14 (0.51–2.51)
Elevated blood pressure & Other components	0.34 (−0.28–0.97)	0.23 (− 0.25–0.72)	3.54 (0.01–3.68)
Elevated FBG & Other components	0.63 (− 0.34–1.60)	0.38 (− 0.19–0.94)	22.94 (0.01–17.99)

*FBG* fasting blood glucose, *CHD* coronary heart disease, *HR* hazard ratios, *CI* confidence interval, *RERI* relative excess risk due to interaction, *AP* attributable proportion of interaction, *SI* synergy index

^b^ Adjustment for alcohol consumption, smoking status, age, sex and family history of hypertension, family history of diabetes and family history of coronary heart disease

### Association between metabolic syndrome component number and CHD

Table 5 shows that set one component as the reference group, in participants with 3 to 5 components, the HRs of CHD gradually increased, the adjusted HR is 1.94 (1.38–2.74) to 4.28 (2.42–7.56).

**Table 5:** Association between metabolic syndrome component number and coronary heart disease

Number of components	CHD, n (%)	*P*	HR ^b^ (95% CI)
1 component	53 (17.43)		1 (*ref*)
2 components	86 (28.29)		1.28 (0.90–1.80)
3 components	92 (30.26)		1.94 (1.38–2.74)
4 components	40 (13.16)		2.14 (1.41–3.25)
5 components	16 (5.26)	< 0.001	4.28 (2.42–7.56)

*P* for trend test of the 1–5 components. ^b^ Adjustment for alcohol consumption, smoking status, age, sex and family history of hypertension, family history of diabetes and family history of CHD

### The association between different combinations of metabolic syndrome components and CHD

The components of *metabolic syndrome* in diagnosed patients could be amalgamated into 15 different combinations. When the number of components was 3, high TG+ low HDL-C + elevated blood pressure was taken as the reference group. We found large waistline + elevated FBG+ high TG (adjusted HR 2.37; 95% CI 0.43–12.99), large waistline + elevated FBG+ low HDL-C (adjusted HR 2.39; 95% CI 0.43–13.12), large waistline + elevated blood pressure + low HDL-C (adjusted HR 1.62; 95% CI 0.58–4.49), large waistline + elevated blood pressure + elevated FBG (adjusted HR 1.57; 95% CI 0.50–4.94), large waistline + elevated blood pressure + high TG (adjusted HR 1.88; 95% CI 0.60–5.84), elevated blood pressure + elevated FBG+ low HDL-C (adjusted HR 2.30; 95% CI 0.62–8.58) had higher risk of CHD, however, there were no significant difference.

When the number of components was 4 and set high TG+ low HDL-C+ elevated blood pressure + large waistline as the reference group. We found high TG+ low HDL-C+ elevated FBG+ large waistline (adjusted HR 4.26; 95% CI 1.43–12.73), and elevated blood pressure + elevated FBG+ low HDL-C+ large waistline (adjusted HR 2.23; 95% CI 1.08–4.59) can significantly increase risk of CHD (Table 6).

**Table 6:** The association between different combinations of metabolic syndrome components and CHD

3	Combinations of metabolic syndrome components	N	CHD, n (%)	*P*	HR ^b^ (95% CI)
	High TG+ low HDL-C+ elevated FBG	17	0 (0)	–	–
	High TG+ low HDL-C + elevated blood pressure	105	4 (3.81)	0.851	1 (*ref*)
	*Large waistline* + elevated blood pressure + low HDL-C	663	12 (1.81)	0.002	1.62 (0.58–4.49)
	*Large waistline* + elevated blood pressure + elevated FBG	130	50 (38.46)	< 0.001	1.57 (0.50–4.94)
	Large *waistline* + elevated blood pressure + high TG	124	11 (8.87)	0.014	1.88 (0.60–5.84)
	Large w*aistline* + elevated FBG+ high TG	19	2 (10.53)	0.166	2.37 (0.43–12.99)
	Large w*aistline* + elevated FBG+ low HDL-C	48	2 (4.17)	0.998	2.39 (0.43–13.12)
	Elevated blood pressure + elevated FBG+ high TG	24	1 (4.17)	0.998	0.83 (0.09–7.47)
	Elevated blood pressure + elevated FBG+ low HDL-C	53	5 (9.43)	0.055	2.30 (0.62–8.58)
	High TG+ low HDL-C+ large *waistline*	194	5 (2.58)	0.260	0.94 (0.25–3.52)
4	Elevated blood pressure + elevated FBG+ Low HDL-C+ high TG	17	0 (0)	–	–
	High TG+ low HDL-C+ elevated blood pressure + large *waistline*	306	17 (5.56)	0.217	1 (*ref*)
	High TG+ low HDL-C+ elevated FBG+ large *waistline*	34	4 (11.76)	0.027	4.26 (1.43–12.73)
	Elevated blood pressure + elevated FBG+ low HDL-C+ large *waistline*	97	13 (13.83)	< 0.001	2.23 (1.08–4.59)
	Elevated blood pressure + elevated FBG+ high TG+ large *waistline*	41	6 (14.63)	0.001	2.20 (0.87–5.59)

*FBG* fasting blood glucose, *HDL-C* high-density lipoprotein cholesterol, *TG* triglycerides, *CHD* coronary heart disease, *HR* hazard ratios, *CI* confidence interval. N is the number of participants in the combination of components. *P* is the result of Chi-square test of different combinations of components and coronary heart disease

^b^ Adjustment for alcohol consumption, smoking status, age, sex and family history of hypertension, family history of diabetes and family history of coronary heart disease

## Discussion

The underlying pathology of CHD is progressive stenosis of the coronary arteries due to arteriosclerosis (AS), which is promoted by inflammation, lipid metabolism disorders, and oxidative stress. A Japanese cohort study found that patients with metabolic syndrome had a great risk of arteriosclerosis, with an odds ratio of 2.07 (95% CI 1.62–2.27) [17]. We found that patients who had metabolic syndrome were 1.81 times more likely to have CHD risk compared to individuals without metabolic syndrome after adjusting for alcohol consumption, smoking status, age, sex, and family history of hypertension, diabetes and CHD. Due to differences in study populations and varying definitions of metabolic syndrome, although the degree of effect of metabolic syndrome in various studies were different, they were all concordant with the present study regard to the finding that metabolic syndrome is a risk factor for CHD. A study comprised of 12,403 patients showed that mandatory diagnostic requirements for (≥ 3) risk factors for metabolic syndrome, which can cause the predictive value of these factors to be lost completely or partly [18]. The present study solidifies the view that metabolic syndrome has a weaker impact on CHD than elevated blood pressure and elevated FBG after adjusting the components using multivariate analysis. It has been pointed out that the presence of metabolic syndrome per se has no better predictive value than some of its components, and this might be attributable to the action of these components as independent risk factors [19].

Metabolic syndrome, as a representative disease, is defined by a group of factors that share common underlying pathological mechanisms. Insulin resistance has an indisputable role in the basis of metabolic syndrome-related pathophysiology; the mechanisms of this adverse effect is acceleration of the production of circulating malondialdehyde-modified low density lipoprotein and impairment of endothelial function [20]. We found that elevated FBG (adjusted HR 1.82; 95% CI 1.38–2.38) increased the risk of CHD, studies have reported that FBG is a better marker of insulin resistance, which might be because inflammation and impaired endothelial function worsen the risk of CHD [18, 21]. Previous research on CHD and the metabolic syndrome components reported different conclusions, mostly in relation to about whether blood pressure plays a pivotal role in the progression of CHD [22, 23]. The present study demonstrated that blood pressure is a sensitive independent predictor of CHD in this population.

One research study on 1548 patients who received elective coronary artery bypass grafting showed that higher HDL-C did not show protective effects in secondary prevention of coronary heart disease [24]. Therefore, there is still some controversy about the mechanism by which a high HDL-C level helps prevent CHD. We did not observe any association between hyper-triglyceridemia and CHD, although epidemiological evidence of the association between elevation of TG and the development of CHD had been confirmed [25], some previous studies were contrary to this association [26, 27]. Furthermore, central obesity is a core component of the diagnostic criteria of metabolic syndrome and a risk factor for chronic diseases. However, there was disagreement on any link between a large waistline, as an indicator of central obesity, and the development of CHD [28, 29]. Despite the present study not found that the above components are related to development of CHD, their effect on CHD cannot be ignored, and observed effect might be related to the optimal cut-offs in this population. Our previous research proved that there was an ideal cut-off point of *waistline* girth for Uighurs [30]. Further research is required to determine whether components defined by different cut-off points have different effects on the risk of CHD in order to obtain more appropriate diagnostic criteria for this population, to better screen high-risk groups, and to promote the prevention of chronic diseases in Xinjiang.

A prospective study on the general Asian population in general confirmed that the presence of coexistent impaired fasting glucose and prehypertension leads to an increase in the incidence of cardiovascular diseases [31]; a German (DIG) study also found this phenomenon for the combination of diabetes and hypertension [32]. On investigating interaction of elevated FBG and elevated blood pressure on CHD, we found a negative multiplicative interaction (multiplicative SI 0.79; 95%CI 0.39–1.58) and a positive additive interaction (additive SI 1.14, 95%CI 0.51–2.51), none of these interactions have significant differences. Studies showed that when biological interactions were discussed, the additive interactions were more credible if multiplicative interactions and additive interactions conflicted [33]. Although there was no significant biological interaction between elevated FBG and blood pressure, there was a positive effect for CHD. That suggested that elevated FBG and elevated blood pressure have independent effects on the development of CHD in this population of Xinjiang.

Current research found that the aggregation of metabolic syndrome components can heighten the risk of CHD. As the number of components increased, there were corresponding increases of adjusted HR of CHD. A prospective cohort of CHD in Iran also obtained this result [7], and a cohort in China reached the same conclusion for cardiovascular disease [22]. When the number of components was the same, this study found that there are different correlations of component combinations with the risk of CHD. Further observed indicate that each combination which increase the risk of CHD involves large *waistline*. Prior studies suggested that participants with abnormal *waistline girth* (≥ 90 cm in males/ ≥ 85 cm in females) are at higher risk for 10-year-CHD (odds ratio = 3.168) in Tibet and Xinjiang [34]. Furthermore, the prevalence of hypertension and obesity in Xinjiang was significantly higher than the national average level [14, 35], and there was a high number of patients with CHD in this study (hypertension 242/304 and central obesity 202/304). Therefore, for primary prevention of CHD in this population, comprehensive prevention measures should be implemented for the management and treatment of elevated blood pressure, elevated FBG and obesity (especially central obesity).

### Limitations

This study has some limitations. First, the cumulative incidence of CHD may have been underestimated because the study did not include patients with CHD events who did not attend local hospitals for various reasons. Second, although we attempted to use impartial questionnaires, bias from self-reporting is inevitable. Third, the measurement of metabolic syndrome did not include all the possible components, such as inflammation, impaired glucose metabolism, insulin resistance, to name a few. This study was a long-term cohort study on CHD conducted in Xinjiang rural populations, and the data, which was obtained from national medical examination records as well as from hospital and social security sources, was reliable and accurate; this fact partly mitigates the above limitations.

## Conclusion

This study showed that the elevated blood pressure and elevated FBG are independent risk factors that increase the risk of CHD. Although our findings suggested that interactions between the metabolic syndrome components do not affect the risk of CHD, their effects on CHD cannot be ignored. The lack of significant biological interaction might be caused by the optimal cut-off points selected, or it might due to insufficient sample size. The *hazard ratios* of CHD increased with the number of metabolic syndrome components. Further observed combinations pointed out that large *waistline* already involves each combination. Therefore, it is important to instigate measures to control blood pressure, blood glucose, and obesity for the prevention of CHD in this population.

## Data Availability

The datasets are available from the corresponding author on reasonable request. The Chinese questionnaire copy may be requested from the authors.

## Abbreviations

CHD: Coronary heart disease
HR: Hazard ratio
CI: Confidence interval
SI: Synergy index
FBG: Fasting blood glucose
TG: Triglyceride
HDL –C: High-density lipoprotein cholesterol
RERI: Relative excess risk due to interaction
AP: Attributable proportion of interaction
